# Accelerated deep learning-based function assessment in cardiovascular magnetic resonance

**DOI:** 10.1007/s11547-025-02019-6

**Published:** 2025-05-17

**Authors:** Domenico De Santis, Federica Fanelli, Luca Pugliese, Giovanna Grazia Bona, Tiziano Polidori, Curzio Santangeli, Michela Polici, Antonella Del Gaudio, Giuseppe Tremamunno, Marta Zerunian, Andrea Laghi, Damiano Caruso

**Affiliations:** Department of Medical-Surgical Sciences and Translational Medicine, School of Medicine and Psychology, Sapienza - University of Rome, Sant’Andrea University Hospital, Via Di Grottarossa, 1035-1039, 00189 Rome, Italy

**Keywords:** Cardiovascular magnetic resonance, Cardiac MRI, Deep learning, Artificial intelligence, Cardiac function, Fast imaging

## Abstract

**Purpose:**

To evaluate diagnostic accuracy and image quality of deep learning (DL) cine sequences for LV and RV parameters compared to conventional balanced steady-state free precession (bSSFP) cine sequences in cardiovascular magnetic resonance (CMR).

**Material and methods:**

From January to April 2024, patients with clinically indicated CMR were prospectively included. LV and RV were segmented from short-axis bSSFP and DL cine sequences. LV and RV end-diastolic volume (EDV), end-systolic volume (EDV), stroke volume (SV), ejection fraction, and LV end-diastolic mass were calculated. The acquisition time of both sequences was registered. Results were compared with paired-samples t test or Wilcoxon signed-rank test. Agreement between DL cine and bSSFP was assessed using Bland–Altman plots. Image quality was graded by two readers based on blood-to-myocardium contrast, endocardial edge definition, and motion artifacts, using a 5-point Likert scale (1 = insufficient quality; 5 = excellent quality).

**Results:**

Sixty-two patients were included (mean age: 47 ± 17 years, 41 men). No significant differences between DL cine and bSSFP were found for all LV and RV parameters (*P* ≥ .176). DL cine was significantly faster (1.35 ± .55 m vs 2.83 ± .79 m; *P* < .001). The agreement between DL cine and bSSFP was strong, with bias ranging from 45 to 1.75% for LV and from − 0.38 to 2.43% for RV. Among LV parameters, the highest agreement was obtained for ESV and SV, which fell within the acceptable limit of agreement (LOA) in 84% of cases. EDV obtained the highest agreement among RV parameters, falling within the acceptable LOA in 90% of cases. Overall image quality was comparable (median: 5, IQR: 4–5; *P* = .330), while endocardial edge definition of DL cine (median: 4, IQR: 4–5) was lower than bSSFP (median: 5, IQR: 4–5; *P* = .002).

**Conclusion:**

DL cine allows fast and accurate quantification of LV and RV parameters and comparable image quality with conventional bSSFP.

**Supplementary Information:**

The online version contains supplementary material available at 10.1007/s11547-025-02019-6.

## Introduction

Cardiovascular magnetic resonance (CMR) is the reference standard imaging modality for a comprehensive evaluation of the heart due to its lack of ionizing radiation and its unique capabilities to combine detailed anatomical and function assessment of the cardiac chambers through cine sequences and to obtain tissue characterization by detecting myocardial edema, infiltration, fibrosis, and perfusion defects [1, 2].

Assessment of myocardial function in CMR is based on 2D balanced steady-state free precession (bSSFP) sequences acquired on four-chamber and two-chamber long-axis views, along with short-axis stacks on both ventricles. These cine sequences are characterized by good temporal resolution [3] and provide excellent signal-to-noise ratio and contrast between the myocardium and blood pool, enabling detailed evaluation of ventricular and atrial size and anatomy, as well as global and regional function [4, 5]. Quantitative functional data for the left (LV) and right ventricle (RV) are computed using dedicated post-processing platforms and are the backbone of CMR.

Despite being widely acknowledged as the reference standard for analyzing cardiac function, traditional bSSFP imaging requires several minutes of scanning, accounting for a considerable part of the whole CMR examination [6] and actively contributing to patient discomfort [7]. Deep learning (DL) is gaining ground in the medical field due to the exponential growth of computational power, holding promises of improving image acquisition, reconstruction, and analysis. In particular, DL applications in cine imaging have been focused on reducing the acquisition time and maintaining adequate accuracy [8–10]. In this regard, a novel DL cine sequence based on a neural network able to generate images from highly under-sampled k-space data was initially tested on a population of children and adults with promising results regarding scan time and image quality [11], recently becoming commercially available. Nevertheless, since its first clinical application, the sequence has been trained on al larger DL model and further refined, and, to the best of our knowledge, its performances have yet to be investigated in a clinical setting.

Therefore, our study aimed to evaluate the diagnostic accuracy and image quality of these novel DL cine sequences for LV and RV functional parameters compared to conventional bSSFP cine sequences.

## Materials and methods

### Patient population

Patients who underwent clinically indicated CMR examinations between January and April 2024 were prospectively enrolled in this single-center investigation. The Institutional Review Board approved the study, and written informed consent was obtained by all patients. Exclusion criteria included inability to obtain informed consent and technical failure due to either a lack of short-axis complete LV and RV coverage or inadequate ECG registration during the examination, neither of which occurred.

### Image acquisition

All CMR examinations were performed on a 1.5 T scanner (GE SIGNA Voyager, GE Healthcare, Waukesha, WI), with the patient lying supine feet-first. High-flexibility 16-channel anterior adaptive image receive coil (AIR™, GE Healthcare) and a wireless Bluetooth ECG and respiratory gating system (WGS-100, Ivy Biomedical Systems, Inc., Branford, CT) were also implemented.

After initial anatomical evaluation obtained through image localizers, a stack of short-axis conventional ECG-gated bSSFP (FIESTA, GE Healthcare) sequences was acquired, covering the entire LV and RV from the base to the apex. Subsequently, to test the novel DL cine sequence (Sonic DL™, GE Healthcare), a second stack of short-axis images spanning the whole LV and RV were obtained; detailed scanning parameters of DL cine and conventional bSSFP cine sequences are reported in supplemental Table 1. CMR examinations continued by acquiring bSSFP cine sequences oriented on two-chamber view, three-chamber view, four-chamber view, and left ventricle outflow tract.

**Table 1:** Patient characteristics (*n* = 62)

Variable	Value
Age (y)*	47.4 ± 16.5
Sex	
Male	41 (66)
Female	21 (34)
Weight (kg)*	78.7 ± 14.8
Height (cm)*	172.1 ± 9.0
Body surface area (m^2^)*	1.93 ± 0.2
Heart rate (bpm)*	62.3 ± 12.3
Risk Factors	
Hypertension	24 (39)
Dyslipidemia	16 (26)
Coronary artery disease	7 (11)
Diabetes	3 (5)
Clinical indication	
Cardiomyopathies	28 (45)
Myocarditis	13 (21)
Myocardial viability	12 (19)
Ventricular arrhythmias	4 (6)
Pericarditis	2 (3)
Cardiac and extracardiac mass	2 (3)
Valvular function	1 (2)

Unless otherwise specified, data are numbers, with percentages in parentheses

^*^Data are means ± standard deviation

For each examination, 0.2 mmol/kg of gadobutrol (Gadovist; Bayer Pharma AG, Leverkusen, Germany) was subsequently intravenously administered with an automated dual-syringe power injector (Medrad MRXperion MR Injection System, Bayer Healthcare, Germany) through an 18-gauge cannula placed in a vein of the antecubital fossa at a flow rate of 3 mL/s, followed by a saline chaser administered at a corresponding flow rate. Ten minutes after gadolinium injection, an inversion time (TI) scout sequence was acquired on the short-axis view at the midventricular level to determine the appropriate TI. Subsequently, 2D Phase-Sensitive Inversion Recovery (PSIR) sequences were acquired in short- and long-axis views.

### DL cine sequence

DL cine is a DL-based acquisition and reconstruction technique consisting of two parts: a) a cine sequence acquiring bSSFP echoes using a sparsely and randomly sampled K-space in phase-encode dimension and incoherently in temporal dimension and b) a neural network-based reconstruction algorithm trained on non-accelerated fully sampled bSSFP cine; the user has control over the acceleration factor and the number of RR intervals to use.

Initially described in 2021 [10, 11], DL cine was optimized in its acquisition, reconstruction, and training components. The current version uses a number of excitation (NEX) of 1 for all RR acquisitions and applies 12 unroll networks to improve image quality. The model incorporates datasets from 1.5 Tesla and 3 Tesla, multiple cardiac views, and multiple coils. Additionally, its integration into the scanner allows short reconstruction time by the graphics processing unit (GPU).

### Acquisition time and image analysis

The acquisition time of both DL cine and bSSFP sequences was automatically provided by the scanner and recorded for each patient.

A board-certified radiologist with 8 years of experience in cardiovascular imaging analyzed the bSSFP datasets during the clinical workflow and the same patient’s DL cine dataset after a time interval of 7 days, to minimize recall bias. During the analysis of the DL cine, the results of the previous segmentation were not available to the reader. All analyses were performed using commercial software (Circle cvi^42^ v. 5.11; Circle Cardiovascular Imaging Inc.; Calgary, Alberta, Canada). End-systolic and end-diastolic frames were identified. Left ventricular endocardial and epicardial contours and RV wall contours were automatically detected by the software from the cardiac base to the apex and manually adjusted by the reader when needed; LV papillary muscles and RV trabeculations were included in the blood pool. The LV and RV outflow tracts were included in the segmentations up to the level of the aortic valve cusps and the pulmonary valve. In case of poorly identifiable limits between atria and ventricles, additional frames of the short-axis cine images, two-chamber and four-chamber views, were reviewed to improve segmentation accuracy. Automatically generated end-diastolic volume (EDV), end-systolic volume (ESV), stroke volume (SV), and ejection fraction (EF) of both the RV and LV, as well as LV diastolic myocardial mass, were recorded for all patients.

### Image quality assessment

Two additional board-certified radiologists with 13 and 11 years of experience in cardiovascular imaging, respectively, blinded to the image reconstruction, independently assessed the image quality of DL cine and bSSFP images based on the following determinants: blood-to-myocardium contrast, endocardial edge definition, and motion artifacts. Grading was performed using a 5-point Likert scale (1: non-diagnostic; 2: suboptimal but still diagnostic for volumetric analysis; 3: adequate; 4: good; and 5: excellent) [12]. Images were evaluated in a randomized order, aiming at avoiding potential recall bias, with no less than a week between the evaluation of DL cine and bSSFP of the same patient, and inter-rater agreement was eventually calculated.

### Statistical analysis

A sample size calculation was performed to determine the appropriate number of participants needed to achieve adequate statistical power. A power of 0.90 and a significance level (α) of 0.05 were targeted. According to Cohen’s guidelines, the expected effect size was estimated to be δ = 0.5, representing a moderate effect [13]. A two-sided paired-samples t test power analysis was conducted in R (R Core Team (2024). R: A Language and Environment for Statistical Computing. R Foundation for Statistical Computing, Vienna, Austria. https://www.R-project.org/) using pwr package (Champely S (2020). pwr: Basic Functions for Power Analysis. R package version 1.3–0, https://CRAN.R-project.org/package=pwr). The calculation indicated that a sample size of 54 participants per group would be required to detect an absolute difference of 3% in left ventricle ejection fraction (LVEF) measurements obtained with DL cine and bSSFP sequences (LVEF in adults men, papillary muscles included in LV volume: 63 ± 6% [14]). To account for potential missing data, the sample size was increased by 10%, resulting in a final target sample size of 60 individuals.

Statistical analyses were performed using commercially available software (IBM Corp. Released 2019. IBM SPSS Statistics for Windows, Version 26.0. Armonk, NY: IBM Corp).

The normality of data distribution was assessed by the Kolmogorov–Smirnov test, and values were consequently compared with the paired-samples t test or Wilcoxon signed-rank test, as appropriate. Continuous variables are reported as mean ± standard deviation, while ordinal data are reported as median and interquartile range (IQR).

The analysis of biases and limits of agreement (LOA) between DL cine and bSSFP sequences was investigated with Bland–Altman plots, with differences expressed as percentages of the values; acceptable LOA was set a priori to 10% [15].

Inter-rater agreement was analyzed by means of weighted Cohen’s κ analysis. Coefficients were interpreted as follows: κ < 0.20, slight agreement; κ = 0.21–0.40, fair agreement; κ = 0.41–0.60, moderate agreement; κ = 0.61–0.80, good agreement; and κ = 0.81–1.0, excellent agreement.

A 2-tailed *P* < 0.05 was considered to indicate a significant difference in the tests performed.

## Results

### Patients characteristics

Detailed patient characteristics are reported in Table 1. A total of sixty-two patients were included; 41 (66%) were male, the mean age was 47.4 ± 16.5 years, the age range was 16–80 years, and body surface area (BSA) was 1.93 ± 0.2 m^2^. A total of 29 of 62 (47%) patients had one or more cardiovascular risk factors, the most frequent was hypertension (24 of 62, 39%), followed by dyslipidemia (16 of 62, 26%). Most patients (28 of 62, 45%) underwent CMR examination for known or suspected cardiomyopathy.

### Acquisition time and cardiac function parameters

The mean acquisition time for DL cine was 1.35 ± 0.55 min for DL cine and 2.83 ± 0.79 min for bSSFP sequences (*P* < 0.001). There were no statistically significant differences between DL cine and bSSFP in both LV (*P* ≥ 0.149) and RV function parameters (*P* ≥ 0.128); descriptive statistics of all LV and RV parameters derived from DL cine and bSSFP are reported in Table 2.

**Table 2:** Left ventricle and right ventricle parameters measured on DL cine and bSSFP sequences

Parameter	DL cine	bSSFP	*P* value
LVEDV	156.3 ± 49.0 mL	155.8 ± 49.5 mL	0.675
LVESV	70.5 ± 47.5 mL	70.9 ± 47.5 mL	0.623
LVSV	86.0 ± 22.1 mL	84.9 ± 21.4 mL	0.234
LVEF	58.0 ± 14.8%	57.4 ± 14.6%	0.410
LVEDM	129.4 ± 42.3 g	127.6 ± 43.0 g	0.149
RVEDV	149.3 ± 41.6 mL	149.8 ± 40.9 mL	0.660
RVESV	71.8 ± 37.2 mL	72.6 ± 37.4 mL	0.287
RVSV	76.8 ± 21.5 mL	75.3 ± 21.8 mL	0.128
RVEF	54.5 ± 13.8%	53.9 ± 14.1%	0.176

Data are means ± standard deviation

bSSFP = balanced steady-state free precession, DL = deep learning, LVEDM = left ventricle end-diastolic mass, LVEDV = left ventricle end-diastolic volume, LVEF = left ventricle ejection fraction, LVESV = left ventricle end-systolic volume, LVSV = left ventricle stroke volume, RVEDV = right ventricle end-diastolic volume, RVEF = right ventricle ejection fraction, RVESV = right ventricle end-systolic volume, RVSV = right ventricle stroke volume

**Fig. 1:**
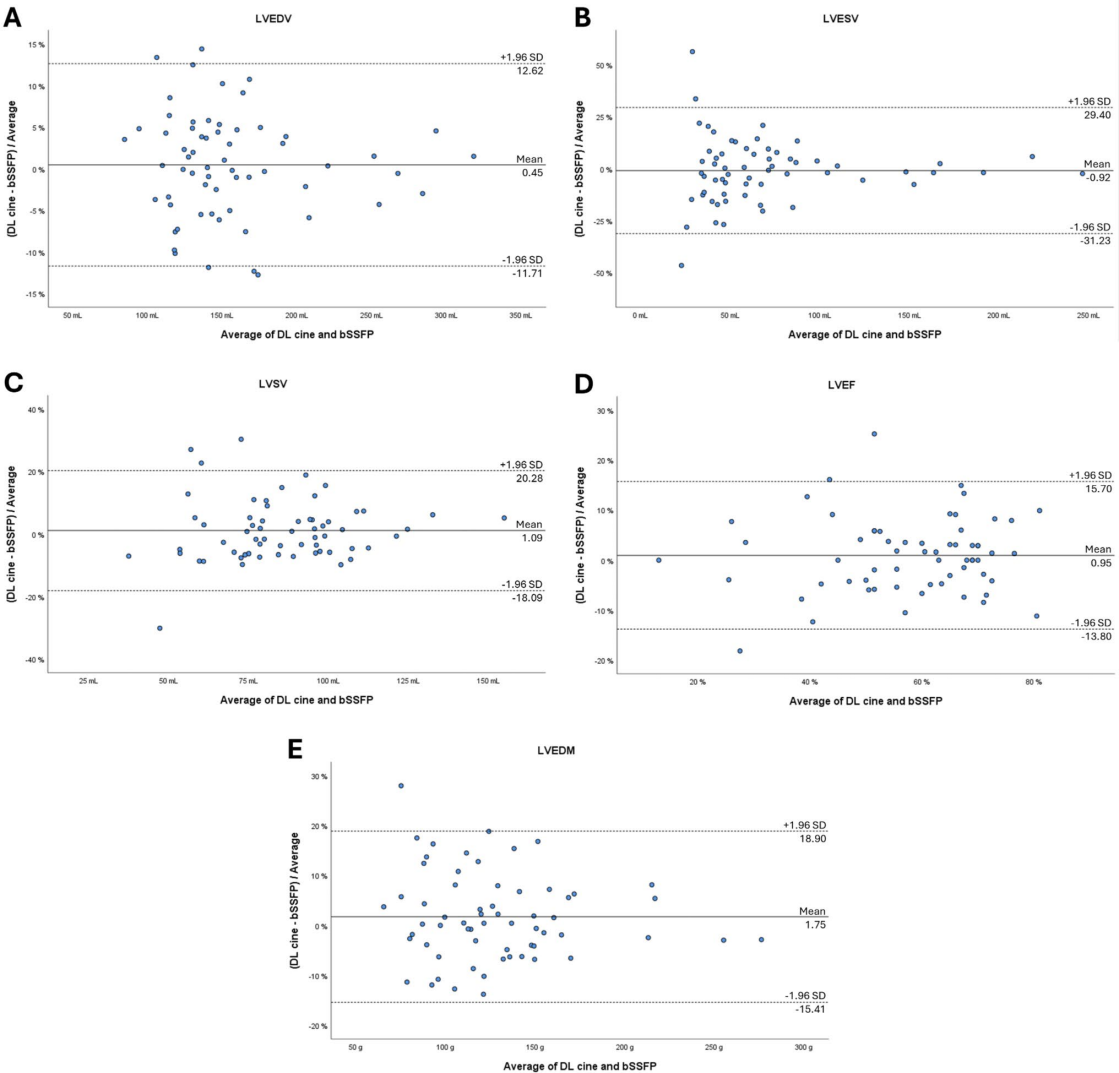
Bland–Altman plots comparing measurement differences in left ventricular function parameters between deep learning (DL cine) and balanced steady-state free precession (bSSFP) cine techniques. Solid lines indicate mean differences; dashed lines correspond to limits of agreement (± 1.96 standard deviations from mean difference); and differences are expressed as percentages of the values. **A**, LV end-diastolic volume; **B**, LV end-systolic volume; **C**, LV stroke volume; **D**, LV ejection fraction; and **E**, LV end-diastolic mass

**Fig. 2:**
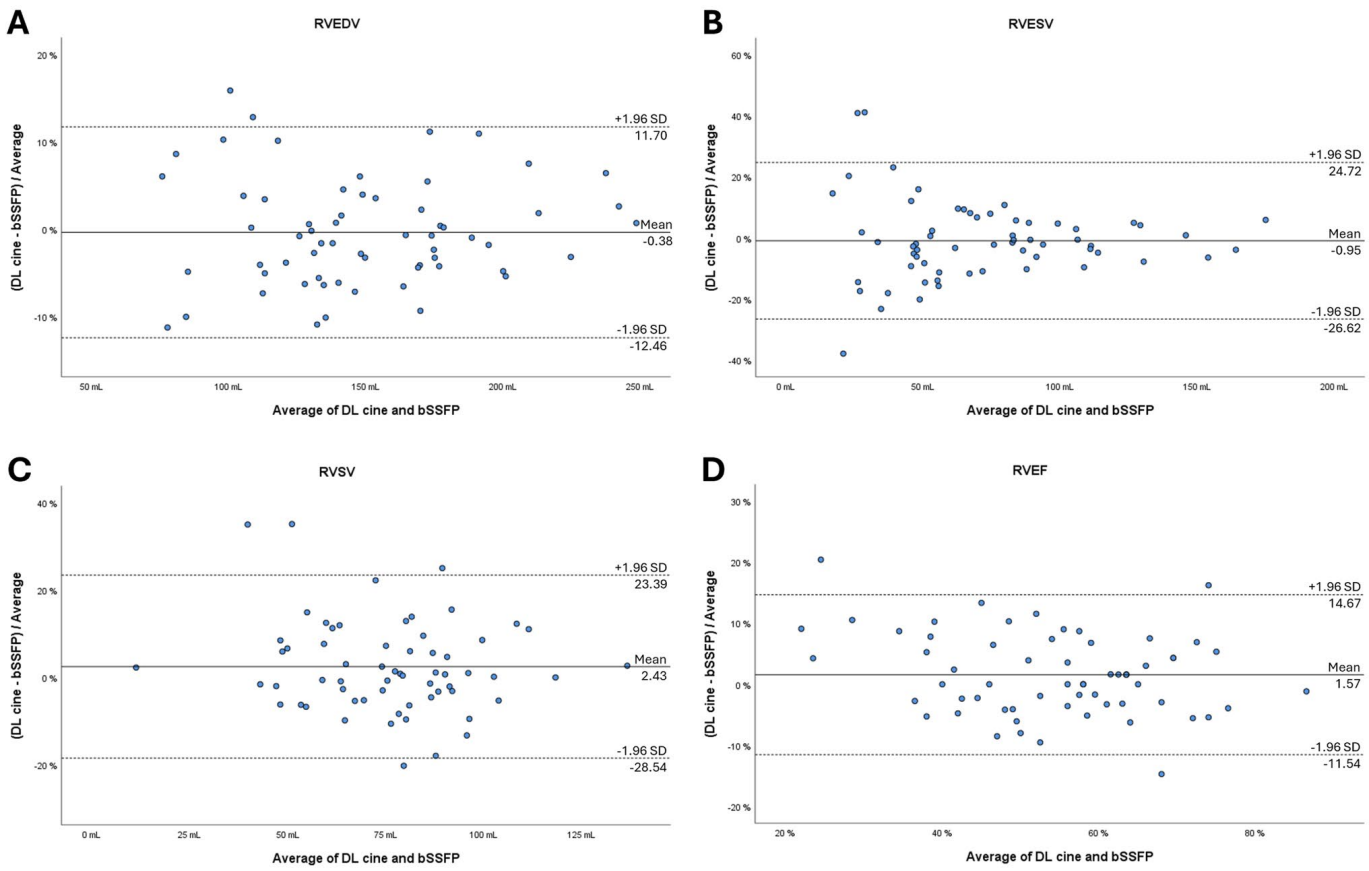
Bland–Altman plots comparing measurement differences in right ventricular function parameters between deep learning (DL cine) and balanced steady-state free precession (bSSFP) cine techniques. Solid lines indicate mean differences; dashed lines correspond to limits of agreement (± 1.96 standard deviations from mean difference); and differences are expressed as percentages of the values. **A**, RV end-diastolic volume; **B**, RV end-systolic volume; **C**, RV stroke volume; and **D**, LV ejection fraction

Differences between DL cine and bSSFP datasets were normally distributed. Bland–Altman plots for LV and RV parameters are shown in Figs. 1 and 2, respectively. Among LV function parameters, LVEDV and LVEF obtained the highest agreement between DL cine and bSSFP, with a bias = 0.45% and 0.95%, respectively, with 57 of 62 (92%) cases falling inside the LOA defined a priori. LVESV and LVSV yielded a bias = -0.92% and 1.09%, respectively, with 52 of 62 (84%) cases inside the acceptable LOA. LVEDM yielded a bias = 1.75%, with 51 of 62 cases (82%) inside the acceptable LOA. Concerning RV function parameters, RVEDV obtained the highest agreement between DL cine and bSSFP, falling inside the LOA defined a priori in 90% (56 of 62) of cases, followed by RVEF (89%, 55 of 62), RVESV (87%, 54 of 62), and RVSV (79%, 49 of 62).

### Subjective image quality

**Fig. 3:**
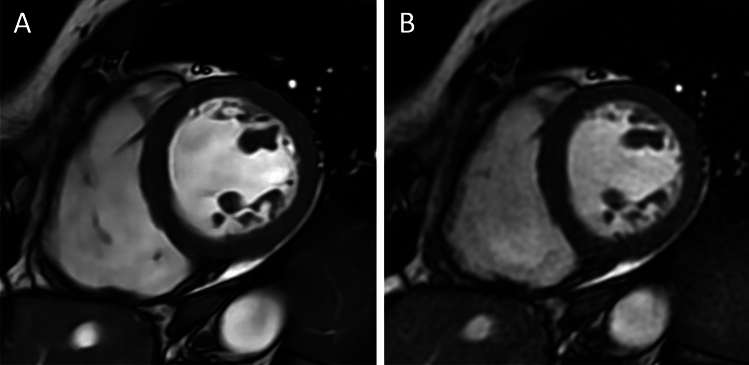
Case example of a 52-year-old female who underwent CMR examination for suspected myocarditis. **A**, short-axis deep learning (DL cine), and B, balanced steady-state free precession (bSSFP) cine images acquired on the short-axis plane, shown in end-diastole, demonstrating comparable overall image quality and slightly less endocardial edge definition of DL cine dataset

**Fig. 4:**
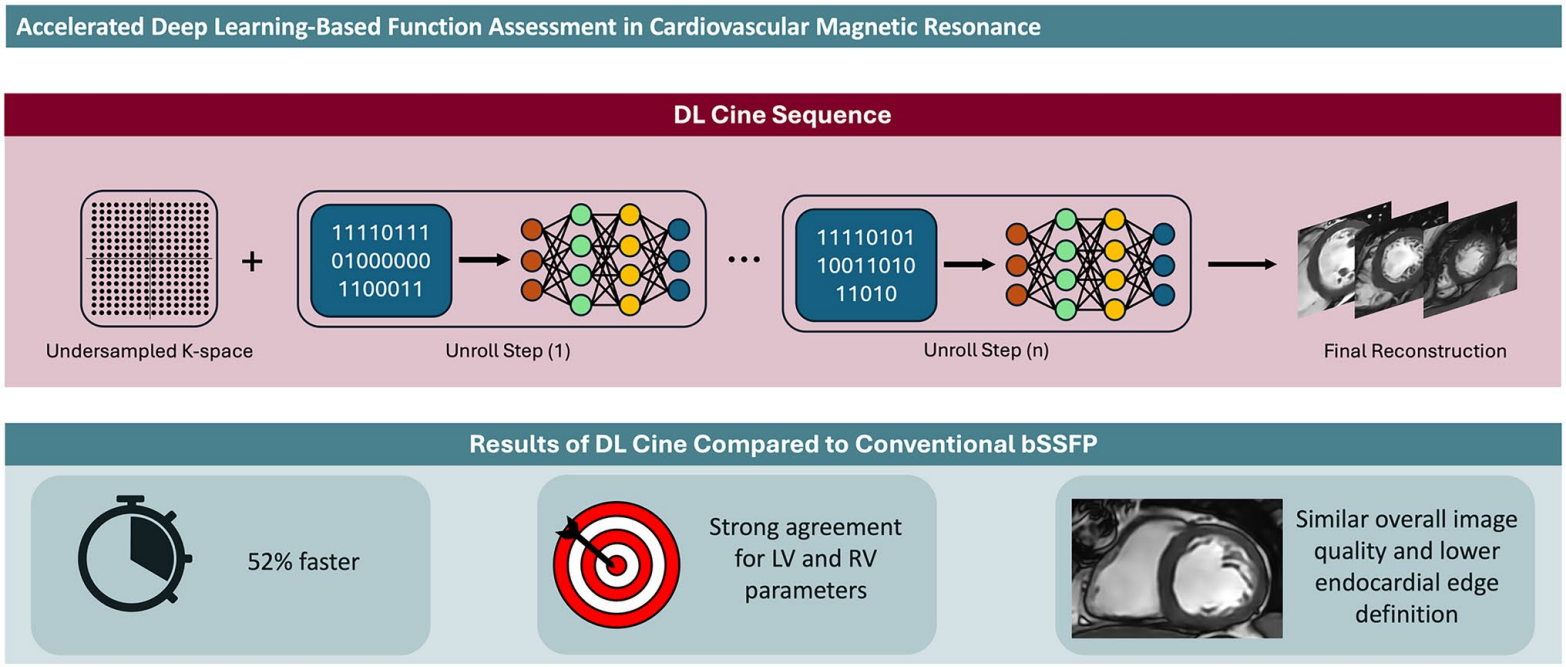
The figure provides a graphical summary of deep learning (DL cine) sequence architecture and the main findings of our study. DL cine was 52% faster than balanced steady-state free precession (bSSFP) sequence, yielded strong agreement for left ventricle (LV) and right ventricle (RV) parameters, comparable overall image quality and lower endocardial edge definition

Comprehensive image quality scores are available in Table 3. Overall subjective image quality was similar between DL cine and bSSFP (median: 5, IQR: 4–5; *P* = 0.330). In particular, blood-to-myocardium and motion artifacts were deemed comparable between DL cine and bSSFP (*P* = 0.739 and 0.064, respectively), while endocardial edge definition of DL cine (median: 4, IQR: 4–5) was rated lower than bSSFP (median: 5, IQR: 4–5; *P* = 0.002), as shown in Fig. 3; side-by-side comparison of bSSFP and DL cine is also available as Supplemental video. An overview of the key findings of the study is shown in Fig. 4. Inter-rater agreement between the two readers was good for both DL cine (κ = 0.736, *P* < 0.001) and bSSFP (κ = 0.730, *P* < 0.001); full details of inter-reader agreement are displayed in Table 4.

**Table 3:** Subjective image quality scores obtained on DL cine and bSSFP sequences

Image quality determinant	DL cine	bSSFP	*P* value
Overall	5 (4–5)	5 (4–5)	0.330
Blood-to-myocardium contrast	5 (5–5)	5 (5–5)	0.739
Endocardial edge definition	4 (4–5)	5 (4–5)	0.002
Motion artifacts	5 (4–5)	4 (4–5)	0.064

Data are medians, with interquartile range in parentheses

bSSFP = balanced steady-state free precession, DL = deep learning

**Table 4:** Inter-reader agreement of subjective image quality scores obtained on DL cine and bSSFP sequences

Image quality determinant	DL cine	bSSFP
Overall	0.736	0.730
Blood-to-myocardium contrast	0.785	0.783
Endocardial edge definition	0.743	0.695
Motion artifacts	0.624	0.678

Data are Cohen’s kappa coefficients (κ)

bSSFP = balanced steady-state free precession, DL = deep learning

## Discussion

Our study aimed to evaluate the diagnostic accuracy and image quality of novel deep learning (DL) cine sequences for left ventricle (LV) and right ventricle (RV) functional parameters, compared to conventional balanced steady-state free precession (bSSFP) cine sequences in cardiovascular magnetic resonance. DL cine sequences yielded comparable LV and RV parameters (*P* > 0.149) and high agreement with bSSFP. DL cine was also 52% faster (*P* < 0.001) and comparable to bSSFP sequences in terms of subjective image quality, except for lower performances in depicting endocardial edge definition.

The implementation of DL in medical imaging is gaining momentum due to the constant increase in computational power, holding the promise of playing a significant role in CMR imaging by enhancing the analysis of cardiac function, structure, and tissue composition. By leveraging deep learning algorithms, researchers can automate tasks such as image segmentation, feature extraction, pattern recognition, and disease classification, ultimately enabling the extraction of diagnostic and prognostic imaging biomarkers and contributing to improved risk stratification and personalized treatments [16–18]. Nevertheless, it has also been demonstrated that DL reconstructions are characterized by intrinsic instability, potentially resulting in various image artifacts even with tiny perturbations of the images. Therefore, caution is required when assessing such datasets [19]. In our investigation, DL cine demonstrated minimal and negligible systematic difference with bSSFP, proved by a bias consistently close to zero in all measured parameters, with no relevant tendency in measurements overestimation or underestimation. The precision of measurements tended to improve in LVEDV, LVESV, LVEDM, and RVESV for higher measurement, while it was evenly distributed in the other parameters across all measurements. DL cine achieved the highest accuracy in measuring EDV and EF of both LV and RV, with 90% or more cases inside the a priori LOA. The lowest accuracy was found in RVSV, in which 79% of cases were inside the a priori LOA; this result might be explained by the low precision of measurements, resulting in a relatively broad data spread and two outliers.

The magnitude of bias and the range of agreement for RV and LV function parameters estimated with DL cine and bSSFP obtained by our investigation is at least comparable or better than previously published data focused on comparing the same datasets [11, 12, 20]. Additionally, being characterized by a bigger patient population, our investigation further strengthens the results of this comparison, highlighting the potential of DL applied to cardiac function parameters. Notably, despite maintaining negligible bias, DL obtained slightly worse agreement performances in assessing RV compared to LV. These results can be explained by the higher anatomical complexity of the RV, mainly due to its crescent shape, thinner wall, and distinctive orientation pattern of its muscle fibers. Moreover, an accurate RV assessment ought to consider its anatomical and functional interaction with pulmonary circulation, as well as its morphological adaptation to preload and afterload [21–23]. Additionally, the importance of accurate RV functional assessment is underlined by the fact that RV parameters might have higher prognostic weight than LV parameters and can predict certain cardiac conditions such as pulmonary hypertension and heart failure [24, 25].

DL cine demonstrated an overall similar image quality to bSSFP. This result is in accordance with a recent investigation comparing conventional cine sequences with DL cine datasets based on a different network architecture [26]. Notably, our results might be perceived as discordant with the ones obtained by Zucker et al. [11], who reported lower image quality for DL cine compared to bSSFP. Nevertheless, it is worth noting that in our investigation, we rated not only the overall image quality but also its determinant, one of which, namely endocardial edge definition, was sensibly lower for DL cine. This result is in accordance with previous literature [12], and despite no direct evidence of over-smoothing in DL reconstructed images in CMR, it might be related to the k-space under-sampling. Nevertheless, the comparable scores in blood-to-myocardium contrast and motion artifacts ultimately accounted for overall similar quality. This characteristic, associated with sensibly faster scan time and good agreement in LV and RV function parameters, might pave the way for a gradual implementation of DL cine in clinical practice, potentially replacing conventional bSSFP cine sequence and ultimately resulting in high-quality, high-diagnostic yield and better tolerated CMR examination.

Some study limitations need to be disclosed. First, this is a single-center investigation performed on a single vendor scanner and focused on a specific DL cine sequence. Therefore, our results might not be fully generalizable to other vendors and DL algorithms. Second, DL cine and bSSFP sequences were only compared in the short-axis plane, while two- and four-chamber views might also be acquired with DL and used to improve the accuracy of ventricular measurements; nevertheless, cardiac volumes are conventionally primarily derived from short-axis image dataset. The third limitation derives from the demographic characteristics of our Institution’s catchment area, wherein pediatric patients are primarily directed to a specialized pediatric hospital in the city. Consequently, our study sample consisted exclusively of adults seeking medical care at our facility, and our results might not be fully transferrable to a pediatric population. However, DL cine has already obtained good diagnostic performance in evaluating young patients with congenital heart diseases [11]. Fourth, we did not assess myocardial wall motion, myocardial strain, and other specific morphological features since they were beyond the scope of our investigation. Further studies are needed to determine the potential of DL cine across the spectrum of specific cardiac diseases.

In conclusion, deep learning (DL) cine imaging demonstrated a considerable reduction in acquisition time and yielded strong agreement with balanced steady-state free precession (bSSFP) in assessing both right ventricle (RV) and left ventricle (LV) function parameters, as well as LV mass. The overall image quality of DL cine is comparable to bSSFP, while efforts are needed to improve myocardial edge definition. By further refinements and optimizations, DL cine holds promise to set new standards for myocardial functional assessment in cardiovascular magnetic resonance, improving clinical workflow and patient tolerance.

## Supplementary Information

Below is the link to the electronic supplementary material.

Supplementary file1 (DOCX 14 KB)

Supplementary file2 (MOV 143719 KB)
