# Supplementary material for: Accelerated deep learning-based function assessment in cardiovascular magnetic resonance
Source: Radiol Med. 2025 May 17;130(8):1149–57. doi: 10.1007/s11547-025-02019-6 (PMC12367930; doi:10.1007/s11547-025-02019-6)
Supplement: Supplementary file 1 — Supplementary file1 (DOCX 14 KB) [file 11547_2025_2019_MOESM1_ESM.docx]

| **Parameter** | **DL cine** | **bSSFP** |
| --- | --- | --- |
| TR (ms) | 3.7 | 4.1 |
| TE (ms) | 1.8 | 1.8 |
| Flip angle (°) | 70 | 70 |
| FOV (mm) | 380 x 380 | 380 x 380 |
| Matrix (pixel) | 224 x 320 | 224 x 224 |
| Spatial resolution (mm) | 1.7 x 1.2 | 1.7 x 1.7 |
| Slice thickness/gap (mm) | 8/0 | 8/0 |
| Bandwidth (Hz/pixel) | 893 | 1116 |
| Views per segment (n) | 14 | 18 |
| Cardiac phases^*^ | 30 | 30 |
| DL = Deep learning, bSSFP = balanced steady-state free precession, FOV = Field of view, TE = echo time, TR = repetition time.  ^*^Retrospective ECG-triggering | | |

**Supplemental Table 1: MRI scanning parameters of DL cine and bSSFP sequences**
